# The Field-Testing of a Novel Integrated Mapping Protocol for Neglected Tropical Diseases

**DOI:** 10.1371/journal.pntd.0001380

**Published:** 2011-11-15

**Authors:** Sonia Pelletreau, Mawuli Nyaku, Massitan Dembele, Boubacar Sarr, Philip Budge, Rachael Ross, Els Mathieu

**Affiliations:** 1 Division of Parasitic Diseases and Malaria, National Center for Global Health, Centers for Disease Control and Prevention, Atlanta, Georgia, United States of America; 2 School of Public Health, University of Alabama, Birmingham, Alabama, United States of America; 3 Ministère de la Santé, Bamako, Mali; 4 Ministère de la Santé, Dakar, Senegal; 5 Epidemic Intelligence Service, Centers for Disease Control and Prevention, Atlanta, Georgia, United States of America; 6 Rollins School of Public Health, Emory University, Atlanta, Georgia, United States of America; London School of Hygiene & Tropical Medicine, United Kingdom

## Abstract

**Background:**

Vertical control and elimination programs focused on specific neglected tropical diseases (NTDs) can achieve notable success by reducing the prevalence and intensity of infection. However, many NTD-endemic countries have not been able to launch or scale-up programs because they lack the necessary baseline data for planning and advocacy. Each NTD program has its own mapping guidelines to collect missing data. Where geographic overlap among NTDs exists, an integrated mapping approach could result in significant resource savings. We developed and field-tested an innovative integrated NTD mapping protocol (Integrated Threshold Mapping (ITM) Methodology) for lymphatic filariasis (LF), trachoma, schistosomiasis and soil-transmitted helminths (STH).

**Methodology/Principal Findings:**

The protocol is designed to be resource-efficient, and its specific purpose is to determine whether a threshold to trigger public health interventions in an implementation unit has been attained. The protocol relies on World Health Organization (WHO) recommended indicators in the disease-specific age groups. For each disease, the sampling frame was the district, but for schistosomiasis, the sub-district rather than the ecological zone was used. We tested the protocol by comparing it to current WHO mapping methodologies for each of the targeted diseases in one district each in Mali and Senegal. Results were compared in terms of public health intervention, and feasibility, including cost. In this study, the ITM methodology reached the same conclusions as the WHO methodologies regarding the initiation of public health interventions for trachoma, LF and STH, but resulted in more targeted intervention recommendations for schistosomiasis. ITM was practical, feasible and demonstrated an overall cost saving compared with the standard, non-integrated, WHO methodologies.

**Conclusions/Significance:**

This integrated mapping tool could facilitate the implementation of much-needed programs in endemic countries.

## Author Summary

Neglected tropical diseases (NTDs) cause significant physical debilitation, lowered economic productivity, and social ostracism for afflicted individuals. Five NTDs with available preventive chemotherapy: lymphatic filariasis (LF), trachoma, schistosomiasis, onchocerciasis and the three soil-transmitted helminths (STH); have been targeted for control or elimination, but resource constraints in endemic countries have impeded progress toward these goals. We have developed an integrated mapping protocol, Integrated Threshold Mapping (ITM) for use by Ministries of Health to decide where public health interventions for NTDs are needed. We compared this protocol to the World Health Organizations disease-specific mapping protocols in Mali and Senegal. Results from both methodologies indicated the same public health interventions for trachoma, LF and STH, while the ITM methodology resulted in a more targeted intervention for schistosomiasis. Our study suggests that the integrated methodology, which is also less expensive and logistically more feasible to implement, could replace disease-specific mapping protocols in resource-poor NTD-endemic countries.

## Introduction

Neglected tropical diseases (NTDs) are parasitic and bacterial diseases that affect an estimated 2.7 billion of the world's poorest people, causing significant physical debilitation, lowered economic productivity, and social ostracism for afflicted individuals [1]. Five NTDs with available preventive chemotherapy: lymphatic filariasis (LF), trachoma, schistosomiasis, onchocerciasis and the three soil-transmitted helminths (STH); have been targeted for control or elimination, but resource constraints in endemic countries have impeded progress toward these goals [2]. In order to achieve the rapid scaling-up of programs necessary to reach elimination and control targets, some by as early as 2020, the global health community is focusing on developing strategies that capitalize on synergies between previously independent elimination and control programs for these diseases. Traditional efforts to treat and prevent NTDs through vertical programs are often costly, and the integration of program components has the potential to cut the costs of NTD programs [3], [4].

At the core of public health efforts to fight the five NTDs mentioned above is a strategy of mass drug administration (MDA) of at-risk populations with safe and effective drugs often donated by pharmaceutical companies [5]. Before an MDA can be launched, a country must demonstrate that the disease threshold for public health intervention, as established by the World Health Organization (WHO), has been surpassed [6], [7]. The first step in this process is to review the available data, followed by collecting missing data by conducting prevalence surveys. Currently, each NTD program has its own methodology [8]. Conducting multiple surveys in the same country can be costly and burdensome to national disease programs. As a result, prevalence surveys are only conducted when funding has been secured, and data to help international partners and program managers with planning and advocacy, and drug donation programs with drug projections are unavailable in many parts of sub-Saharan Africa [8]. For this reason, there is a need for feasible and practical protocols that can be implemented by Ministries of Health and whose results are accepted by WHO and drug donation programs. Because NTDs tend to overlap in geographic areas, it is logical that an integrated approach to mapping NTDs might result in more efficient identification of populations needing treatment [9].

This paper describes a novel methodology, the integrated threshold mapping (ITM) methodology, for an integrated mapping survey for LF, trachoma, schistosomiasis, and STH. Our methodology does not provide prevalence figures, its intended purpose is to determine whether a disease has attained the threshold for public health intervention by attempting to balance epidemiologic rigor and field practicality. The ITM protocol has been field tested in one district in each of two countries by comparing the ITM methodology to the standard disease-specific WHO mapping methodologies. Results from both methodologies were compared in terms of public health intervention based on the disease specific data and feasibility, including cost.

## Materials and Methods

The ITM methodology is derived from rapid mapping methodologies used by LF and schistosomiasis programs. It is designed to provide programmatically useful mapping data in a logistically practical and cost-efficient way.

### Study sites

In Mali, the research site included nine sub-districts of the Banamba district (Koulikoro region), an area with an estimated population of 88,232 persons (2008). In Senegal, research was conducted in all 11 non-urban sub-districts of Diourbel district (Diourbel region), an area with an estimated population of 150,889 persons (2010). In both countries, the majority ethnic groups, Bambara and Wolof, respectively, are subsistence farmers living in established settlements; a minority Peuhl group in Mali is semi-nomadic and engages in cattle herding. The entirety of both the Banamba and Diourbel districts is located within the Soudanian ecological zone [10]. NTD program activities including the provision of preventive chemotherapy for the five NTDs have been ongoing in Banamba district, but not in Diourbel district.

### Indicators and target age groups

The standard indicators and target age groups currently recommended by the WHO for the targeted NTDs were employed for both the ITM and WHO methodologies, although some minor adaptations were made for trachoma Table 1. For LF, the immunochromatography card test (ICT, BinaxNOW Filariasis, Alere Inc.), which tests blood drawn by finger stick, was used to measure the presence of *Wuchereria bancrofti* antigen in persons aged ≥15 years and resident in the village for at least ten years [11]. Results were read after ten minutes and recorded by a trained laboratory technician. For trachoma, an ophthalmic technician examined both eyes of children aged 1–9 years for clinical signs of active trachoma (trachomatous inflammation follicular [TF]) and of females aged ≥15 years for clinical signs of trichiasis (trachomatous trichiasis [TT]) using a binocular loupe and natural light according to the WHO Simplified Grading System [12]. For *Schistosoma haematobium*, the presence of haematuria in urine samples from children aged 6–9 years was detected by urine reagent strips. Reagent strips were dipped in the urine samples and read after one minute by comparing them to a colorimetric scale by a trained laboratory technician. At the request of the Ministry of Health (MoH), urine samples were also tested by filtration in Senegal as part of the WHO methodology [13]. For *Schistosoma mansoni* and STH, the prevalence and egg load (eggs per gram of feces) were calculated using the Kato-Katz method [14].

**Table 1:** Indicators, tests, thresholds and interventions recommended by the World Health Organization[6], [12].

Disease	Indicators	Test	Threshold for Intervention	Public Health Intervention
**Trachoma**				
	Trachomatous Follicular (TF)	Clinical Examination	Prevalence of ≥10% in children aged 1–9 years	MDA in total population
			Prevalence of 5–9% in children aged 1–9 years	Targeted treatment at the sub-district level
	Trichiasis (TT)	Clinical Examination	Prevalence of ≥1% in adults aged ≥15 years	Surgery program
**Schistosomiasis**				
*S. haematobium (S.h)*	Hematuria	Urine dipsticks/filtration	Prevalence of *S.h* and *S.m* between 10–50% in children aged 5–14 years	MDA in school-age children every two years
*S. mansoni (S.m.)*	Eggs in stool	Kato-Katz	Prevalence of *S.h* and *S.m* ≥50% in children aged 5–14 years	MDA in school-age children and high risk population
**Soil-transmitted Helminthes**				
	Eggs in stool	Kato-Katz	Prevalence between 20–50% in children aged 5–14 years	MDA in school-age children
			Prevalence of ≥50% in children aged 5–14 years	MDA twice a year in school-age children
**Lymphatic filariasis**				
	Antigen	ICT test	Prevalence of ≥1% in adults ≥15 years	MDA in total population

### Integrated Threshold Mapping protocol

#### Sampling

**Figure 1:**
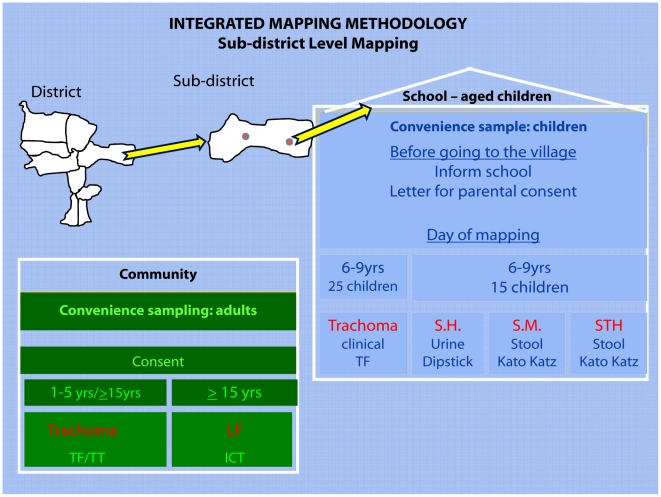
Diagram showing the sampling methodology for the Integrated Threshold Mapping (ITM) Methodology.

**Figure 2:**
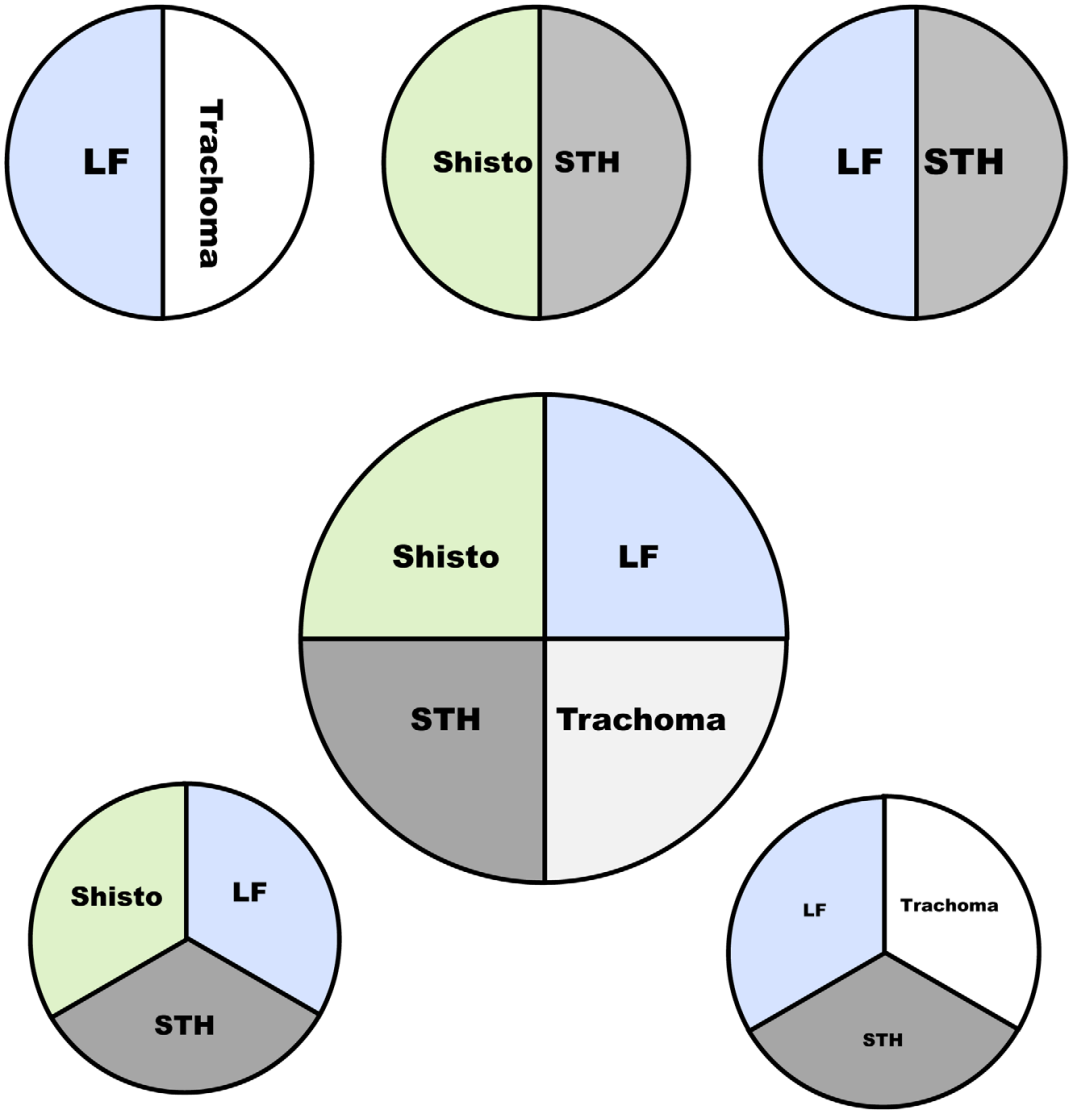
Some of the possible combinations for the Integrated Threshold Mapping Methodology (ITM).

The sampling, as shown in Figure 1, is a combination of random and targeted sampling. Although the protocol is designed for four NTDs, each disease consists of its own module which can be included in the final mapping protocol, depending on the NTD(s) that need mapping in a specific area Figure 2. Two villages per sub-district were selected for trachoma, STH and schistosomiasis testing. One village was randomly chosen, and the other was selected based on suspected high *S. haematobium* prevalence by the district health officer or local dispensary staff, according to information on high haematuria prevalence or the presence of a nearby body of water [15]. When the random village was the same as the village determined to have a high likelihood of schistosomiasis, another village was randomly selected. For LF, only two of the selected villages in the whole study area were included, with the main selection criterion that they were 50 kilometers apart [16].

In the village, the team established a testing site at a school or other central location. A convenience sample of 25 school children aged 6–9 years was selected to be examined for TF, and 15 of these children were also asked to give urine and stool samples. An effort was made to achieve a gender balance. Females aged ≥15 years were asked to come to the central site with their children aged 1–5 years. A convenience sample of 50 of these females was examined for TT, and a convenience sample of 25 children aged 1–5 years was examined for TF, for a total of 50 children tested for TF in each village. In the two villages where LF was surveyed, a convenience sample of 100 persons ≥15 years was tested by ICT. This normally included the 50 females examined for trachoma as well as an additional 50 persons. All persons tested for LF had resided for at least ten years in the village.

#### Logistics

An integrated central team of five health workers from the national level of the MoH conducted the survey. The team included one laboratory technician for LF, two ophthalmic technicians for trachoma and two laboratory technicians for schistosomiasis and STH. Depending on the local situation, the team engaged health staff from the district laboratory to help performing Kato-Katz examinations. At the village level, village chiefs and community health workers were also involved in registration procedures and other logistics. The central team received a half-day training on study methodology, sampling, data collection tools, and the process of obtaining consent and assent. Because the team members were all active in the national programs, are engaged in laboratory work on a daily basis and had been involved in earlier prevalence surveys for these diseases, they were already trained in their respective diagnostic procedures. The CDC team supervised all data collection, laboratory preparations and slide readings for LF, schistosomiasis, and STH to ensure that all procedures were conducted according to the approved protocols. For trachoma, all ophthalmic technicians had been trained in the WHO grading system for trachoma and the examination of patients for trachoma was also part of their daily work.

The team traveled together in one vehicle and visited two villages in one sub-district each day. Specimen collection, point-of-care testing and trachoma examinations were carried out during the day, and the Kato-Katz was performed each evening. In Diourbel, the team perfomed the laboratory testing at the district hospital; in Banamba, the team traveled with all necessary equipment, including miscroscopes and a generator, and a laboratory was set up in the dispensary. The CDC personnel travelling with the team kept records of the time it took to travel to a village, perform the survey, time taken to prepare and read the Kato-Katz and urine samples in the laboratory and time to travel to villages.

### WHO protocol

#### Sampling

For trachoma, the standard WHO 30-cluster population-based prevalence survey was conducted [12]. Thirty clusters were selected using probability proportional to estimated size sampling. Households were selected using an improved EPI random walk and children aged 1–9 years were examined for TF and females aged ≥15 years for TT [17]. Testing continued until the sample size of 80 children and 50 females per cluster was reached. When the required sample size in a village was not attained, the selection continued in an adjacent village. In villages where examinations had already been conducted using the ITM methodology, the team examined only an additional 30 children to avoid examining children twice. For schistosomiasis and STH, the team randomly selected five villages per ecological zone [15]. The number of ecological zones in the district was defined by the national schistosomiasis program coordinator. In each village, the team established a central testing site and took urine and stool samples from a convenience sample of 50 children aged 5–14 years. LF was not reassessed because the ITM methodology used the standard WHO mapping methodology [18].

#### Logistics

The standard WHO mapping protocols were conducted by separate teams for schistosomiasis/STH and trachoma. The teams worked independently. For trachoma, the number of teams was determined by the standard national trachoma program practices. In Mali, two trachoma teams, each composed of two ophthalmic technicians, conducted the trachoma cluster survey. In Senegal, three teams, each composed of one ophthalmic technician, conducted the trachoma cluster survey. A laboratory team, composed of the same two technicians who had conducted the ITM methodology, implemented the WHO methodology for schistosomiasis and STH. A half-day training was provided to all team members on the WHO sampling methodologies, data collection tools, and the process of obtaining consent and assent. No diagnostic training was given because the team members were all active in the national programs as mentioned above.

The WHO standard methodologies were conducted immediately following the completion of the integrated methodology. Each of the three teams traveled in its own vehicle. Testing and examinations were carried out during the day, and the Kato-Katz was performed each evening in the district laboratory or in the dispensary.

### Ethical considerations

The study protocol received ethical approval from the Ethics Committee of the Faculty of Medicine, Pharmacy and Odontology-Stomatology, University of Bamako, Bamako, Mali; from the National Ethics Committee for Health Research (CNERS), Dakar, Senegal; and from CDC's Internal Review Board, Atlanta, GA, USA.

All persons over the age of eighteen years were asked to provide written consent to participate in the study. The school director or a member of the team explained the study to the school-aged children (5–14 years) using a verbal assent script describing the study, and the children who participated in the study were asked to give their verbal assent. Written consent to participate in the study was also obtained from parents or custodians of children between the ages of 1–18 years.

Children who were found to have active trachoma received two tubes of tetracycline ointment, to be applied twice daily for a period of six weeks. Females found to have trichiasis were referred to the nearest health center that provides trichiasis surgery. Persons who tested positive for *W. bancrofti* antigen and children who were found to be infected with *S.haematobium*, *S.mansoni* or STH were referred to the district health center in case an MDA was not planned in the district.

### Feasibility data

To determine feasibility, we took into consideration time and resources needed to conduct field activities and overall costs. All receipts were collected, and actual expenditures were recorded. The cost data were compiled into four categories: training, per diems (national and district levels including lodging), travel to the field (renting of vehicles, drivers and fuel) and supplies (medical and laboratory). Any costs of inputs that were used for multiple activities were distributed evenly among the activities. Although LF testing was only conducted once, costs for conducting the testing were included for both methodologies. The training of health personnel on diagnostic methods, salaries and data entry and analysis conducted by CDC were not included in the cost analysis.

### Data analysis

Results for trachoma, STH and LF were analyzed at the district level for both methodologies Table 2. Results for schistosomiasis were calculated at the sub-district level for the ITM methodology and at the ecological zone level for the WHO methodology. For the ITM methodology, the results of the two villages per sub-district were combined, and for the WHO methodology, all schools were combined. The public health interventions based on the WHO thresholds Table 1, were compared for both methodologies. Feasibility, including cost, time and the human resource needs was compared for both methodologies.

**Table 2:** Implementation Unit (IU) and sampling frame indicated by WHO guidelines and the Integrated Threshold Mapping methodology.

	WHO		ITM	
	IU for Public Health intervention	Mapping sampling frame	IU for Public Health intervention	Mapping sampling frame
**Lymphatic filariasis**	District	District	District	District
**Schistosomiasis**	District/Village	Ecological Homogeneous Area	Sub-district	Sub-district
**Soil-transmitted helminths**	District/Village	Ecological Homogeneous Area	District	District
**Trachoma**	District/Sub-district	District	District/Sub-district	District

## Results

**Figure 3:**
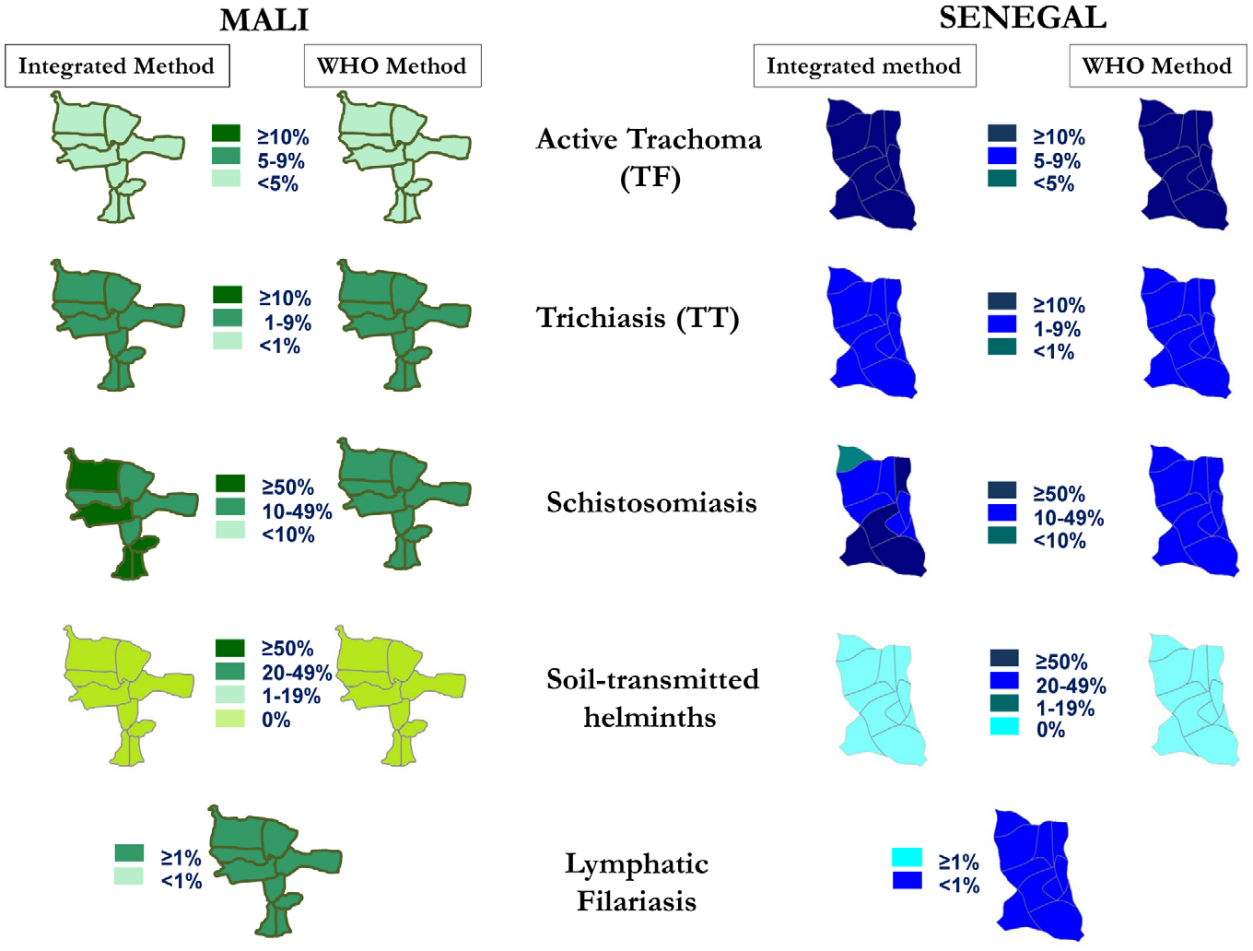
Results from Mali and Senegal from the Integrated Threshold Mapping (ITM) and WHO mapping methodologies.

In Banamba District, Mali, 1,898 persons, including 900 children (1–9 years), in 18 villages were surveyed for the ITM methodology, and 4,479 persons, including 2,738 children (1–9 years) in 35 villages were surveyed for the WHO methodology. For trachoma, eight villages already tested for the integrated method were also sampled for the WHO method. As shown in Figure 3, the results of both methodologies indicated no need for MDA for trachoma (ITM Method: 1.9%, WHO method: 4.7%, 95% CI: 2.9–6.5 or STH: 0%, 0%) within the surveyed district. The methodologies were also concordant in indicating a need for TT surgeries (ITM Method: 2.6%, WHO Method: 3.7%, 95% CI: 2.4–5.1). The LF mapping was added to validate the feasibility of the integrated mapping and not to validate the results.

Because the ITM method had as its purpose to refine the schistosomiasis mapping, the two methods differed, in indicating a need for schistosomiasis treatment: the WHO methodology indicated a need for MDA for schistosomiasis among only school-aged children in the entire area. The ITM methodology indicated that four sub-districts were in need of MDA for schistosomiasis among school-aged children only, and that five sub-districts were in need of MDA for schistosomiasis for the whole population. Treating the whole population in the sub-districts where this was indicated by the integrated mapping protocol would result in nearly 33,500 additional at-risk persons being treated than if only school-aged children in these sub-districts were treated (as indicated by the WHO protocol).

In Diourbel District, Senegal, 2,734 persons, including 1,100 children (1–9 years), in 22 villages were surveyed for the ITM methodology, and 4,614 persons, including 2,914 children (1–9 years) in 34 villages for the WHO methodology. For trachoma, one village already selected for the ITM method was also sampled for the WHO method. Both methodologies indicated the need for MDA for trachoma in the surveyed district (ITM Method: 14.9%, WHO Method: 13.4%, 95% CI: 9.8–17.0) and both methodologies indicated that there was no need for intervention for STH: 0%, 0%.

Both ITM and WHO methodologies were also concordant regarding the need for TT surgeries (ITM Method: 4.7%, WHO Method: 6.1%, 95% CI 4.4–7.7). As mentioned above, the LF mapping was added to validate the feasibility of the integrated mapping and not to validate the results.

In Mali, however, the public health interventions differed with regards to schistosomiasis treatment because the ITM method had as its purpose to refine the schistosomiasis mapping. The WHO methodology indicated a need for MDA for schistosomiasis treatment among only school-aged children in the entire area, while the ITM methodology indicated six sub-districts in need of MDA for schistosomiasis among school-aged children only, four sub-districts in need of MDA for schistosomiasis for the whole population and one sub-district which did not pass the threshold for treatment. Compared to the district based WHO protocol, the ITM methodology targeted nearly 46,000 extra at-risk individuals for treatment.

In the ITM methodology, we compared the public health intervention decisions for schistosomiasis that would result from using only the randomly or only the targeted selected village Table 3. Only in nearly half of the sub-districts (5/11 in Senegal; 5/9 in Mali), basing a treatment decision on sampling in a village considered highly endemic for schistosomiasis would have resulted in treatment for more persons than if the decision had been based on sampling in a randomly selected village. The contrary was the case for 3/11 villages in Senegal and 1/9 villages in Mali.

**Table 3:** Public health interventions for the Integrated Threshold Mapping methodology based on various village selections.

	Public health intervention based on village with suspected high schistosomiasis prevalence	Public health intervention based on randomly chosen village	Public health intervention based on the 2 villages combined
**Senegal**			
Ndindy	MDA SAC*	No MDA	MDA SAC
Ngohe	MDA total population	MDA total population	MDA total population
Ndoulo	No MDA	MDA total population	MDA SAC
Thiobe	MDA SAC	No MDA	MDA SAC
Gade Escale	MDA SAC	No MDA	No MDA
Toure Mbonde	MDA total population	MDA total population	MDA total population
Keur Ngalgou	MDA SAC	MDA total population	MDA SAC
Patar	MDA total population	No MDA	MDA SAC
Taiba Moutoupha	MDA total population	MDA SAC	MDA SAC
Tocky Gare	MDA SAC	MDA total population	MDA total population
Danke Sene	MDA total population	MDA total population	MDA total population
**Mali**			
Madina Sacko	MDA total population	MDA SAC	MDA total population
Touba	No MDA	MDA SAC	MDA SAC
Kerouane	MDA total population	MDA total population	MDA total population
Kiban	MDA total population	MDA SAC	MDA total population
Ouleny	MDA total population	MDA SAC	MDA SAC
Toubacoro	MDA total population	MDA total population	MDA total population
Boron	MDA SAC	MDA SAC	MDA SAC
Ouaro	MDA total population	MDA SAC	MDA total population
Guessene	MDA total population	No MDA	MDA SAC

*SAC: school-aged children.

To compare the feasibility of the two protocols, we compared the time it took to conduct each survey. For the ITM methodology, the total number of days on the field was 46 person-days in Mali and 56 persons days in Senegal. It took approximately two hours to survey one village; in a village where LF testing was done, four hours were needed. The schistosomiasis and STH team spent an additional three hours each day preparing and reading the Kato-Katz slides. The time to travel to the villages from the health center of the sub-district varied according to geography from approximately 15 minutes to 1 hour. For the WHO methodology, the total number of days on the field was 56 person-days for Mali and 58 person-days for Senegal. Each trachoma team took approximately 4–5 hours to survey one village. The schistosomiasis and STH team surveyed one village a day with approximately two hours to collect the samples and an additional five hours to prepare and read the 50 Kato-Katz and urine samples in the district laboratory. The time to travel to the villages varied according to geography from approximately 15 minutes to over 2 hours.

The results of the cost data analysis Table 4, indicate that using the ITM methodology resulted in a 31% overall cost savings in Mali ($6,968 vs. $10,039), and a 19% overall cost savings in Senegal ($8,442 vs. $10,372). In both countries, the ITM methodology used resources more efficiently than the WHO methodology in the areas of travel, supplies and team training, as shown in Table 4.

**Table 4:** Financial resources used in the Integrated Threshold Mapping (ITM) and WHO methodologies.

Budget Line Items	Cost by country and methodology									
	Mali					Senegal				
	WHO		ITM		Difference	WHO		ITM		Difference
	USD	%	USD	%	%	USD	%	USD	%	%
**Training**	233	2	167	2	−28	596	6	548	6	−8
**Per diems**	2,106	21	1,763	25	−16	2,378	23	2,671	32	+12
**Travel to the field**	4004	40	1796	26	−55	3235	31	1519	18	−53
*Fuel*	1,544		752			458		297		
*Car rental*	2,193		933			2,222		978		
*Driver(s)*	*267*		*111*			*555*		244		
**Supplies**	3,696	37	3,242	46	−12	4,163	40	3,704	44	−11
**TOTAL**	10,039		6,968		−31	10,372		8,442		−19

## Discussion

We describe an integrated NTD mapping methodology (Integrated Threshold Mapping) that can be used as an operational tool by Ministries of Health in NTD-endemic countries to determine if the threshold needed to launch disease-specific public health intervenions has been reached. With the recent increased interest in NTDs, there is a real need for an integrated mapping approach that can provide district level data in a timely manner [9], [18], [19]. In developing this ITM methodology, we sought to balance epidemiologic rigour with field practicality, resulting in an approach that can be used only for determining where public health interventions are needed. With the exception of some minor modifications for trachoma, we have retained the key indicators and age groups used in the disease-specific WHO mapping guidelines, but have adapted the sampling methodologies. This ITM methodology reduces costs, and the need for manpower and resources, and gives MoHs a simpler and quicker way to estimate their NTD needs.

The first step in making a NTD action plan is to evaluate the existing data, including the methodology and time of the data collection, and to determine whether any factors that could influence the prevalence of the disease have changed since the data were collected. The second step is to collect data where existing data are missing or out of date; our ITM mapping protocol was designed for this purpose. Because it will be rare that all four NTDs will need mapping in any one district, the methodology consists of disease-specific modules. For each specific situation, modules can be combined to create the situation-specific NTD integrated protocol Figure 2.

To understand the value and limitations of this protocol, it is critical to understand that this protocol is not claiming to provide epidemiologically correct prevalence data because both the villages and the individuals tested were not randomly selected. First, this implies that we cannot recommend using findings from this methodology as a baseline for measuring impact at the district level. This is also not possible for the WHO recommended mapping protocols for schistosomiasis, STH and LF, but it is the case for the trachoma WHO methodology. A possible second implication could be that the WHO recommended threshold could not be used when using this methodology; but here again, this would only be an issue for trachoma, bearing that the WHO methodology does not provide epdemiological correct prevalence figures for schistosomiasis, STH and LF.

One barrier to integrated mapping of NTDs is the misconception that mapping can only be integrated if all testing is done using the same age groups. In our ITM mapping protocol, we insisted on using the program-specific age groups as indicated by WHO with the exception of some adaptation for trachoma. The advantage of using these established age groups was that the WHO thresholds were applicable and that few people had to undergo multiple tests and examinations; only in a limited number of cases were people asked to undergo more than one test or examination. In each village, we chose to use a convenience sample for reasons of field practicality. Asking people of different ages to come to a central location in each village facilitated data collection and was much more time-efficient than house-to-house visits, such as those used in the WHO trachoma protocol. As the field testing in both countries showed, the data of both methodologies resulted in the same public health recommendations.

The ITM protocol maintains the WHO-recommended disease indicators and thresholds for LF, schistosomiasis and STH, but we used a novel sampling frame for more practical field implementation. To improve the representativeness for the TF indicator, we selected a pre-determined number of children between the ages of 1–5 and 6–9. This was intended to prevent biased estimates in case the 6–9 year age group made up the majority of children being graded for trachoma because prevalence rates are highest in 1–5 year olds. Although current WHO guidelines measure trichiasis in adults of both sexes as a standard indicator, literature shows that different adult age groups have been used to determine TT prevalence [20], [21], [22]. After consultation with trachoma experts, we decided to use women above the age of 15 yrs [22].

Because the ITM methodology is focused on public health action, the sampling frame is directly linked to the implementation unit for MDA for each of the NTDs. Based on field experience, we decided to slightly modify those MDA implementation units to make public health interventions more feasible in the field, as shown in Table 2. Because a village-centered approach is difficult for implementation and supervision for a large-scale national program, we decided to use the district as the primary implementation unit for STH as it is recommended by WHO for LF and trachoma. Our methodology found the same public health intervention for trachoma and for STH. For STH, both methodologies concluded that no intervention was necessary, which confirmed the reliability of our methodology in the two countries. The LF testing was included to validate only the field feasibility of integrateing testing for four NTDs. In the case of schistosomiasis, a disease that is very focal in nature, and for which treatment medication is costly and in limited supply, we feel that it would be more appropriate to use the sub-district as implementation units to determine needs for treatment because a district-based approach may result in overtreating or undertreating subpopulations within the district [23]. Our results show that by using the sub-district as I.U., the MDA will be more targeted to people at risk. In our study, when the WHO-recommended ecological zone was used as the implementation unit, highly endemic (prevalence of >50%) or low endemic (prevalence of <10%) sub-areas of the district were not identified, but were identified by the integrated methodology. As a result, using the WHO protocol would have meant that tens of thousands of people living in highly endemic areas would not have received treatment, and that other people not in need of treatment would have been treated, as would have been the case in Senegal.

The integrated approach proved to be more efficient in cost, transport, time in the field and the use of human resources. Because transportation and per diems are variables that significantly increase the cost of mapping, we created one small team of experienced technicians already active in the national MoH program instead of using multiple disease-specific teams. We decided to limit the number of team members to five by capitalizing on their laboratory expertise in parasitic diagnostic techniques mastered during their lab technician training. Each member of the team performed multiple tasks, which increased time and energy efficiencies and also made planning and field organization easier. We also counted on the strong engagement of local staff as we saw this as capacity building of district, dispensary, and village health staff. Although the team members from the different NTD programs were initially reluctant to work as one team, because it was a new concept, this hesitancy evaporated over the course of the survey. The team members also noticed that resources were used more efficiently compared to the WHO methodologies: the ITM methodology used one vehicle to transport the team, versus 3 used in the WHO methodologies, employed a 5-member team instead of a 7-member team, and took about half as much time to implement as the WHO methodologies.

It is also important to mention that certain cost savings from integrated mapping such as time saved by only having to plan one survey compared with several different protocol meetings and logistic preparations is priceless for overburdened health staff. In addition, the workload for all the preparations and supervision can be shared among different program coordinators.

It is worth mentioning that cost savings are just a single variable that justifies conducting integrated surveys: mapping is often the first field activity of an integrated NTD program, and the creation of the team demonstrated the concept of integrating vertical programs to all levels of the MoH and this first activity can help with further collaboration for implementing integrated public health interventions.

Although the ITM methodology resulted in substantial cost savings compared to the WHO methodology, the cost depends on the number of sub-units in a district. This means, for example, that the sampling size for schistosomiasis could be much higher using the ITM methodology than using the WHO methodology. However, this would result in a more targeted MDA for schistosomiasis, which is important because there is limited availability of donated praziquantel.

The results from both the ITM and WHO methologies indicate that health workers are not always well-informed about where schistosomiasis is most prevalent. The results show that treatment decisions, based on the purposely-selected villages, did not systematically result in more treatments than those based on the randomly-selected villages.

We encountered some limitations in conducting the integrated mapping. We are comparing a convenience sampling for schistosomiasis and STH mapping with an accepted WHO convenience sampling. Ideally, we would have compared both methods to an independent, “gold standard” survey methodology. The main limitation for the trachoma mapping is that a total of nine of the 60 trachoma villages sampled were selected for both methodologies. To decrease the burden of the inhabitants by not having the same children examined twice, we included the data collected in the integrated mapping for the WHO method and added only 30 children by visiting the households. For three villages, the selection bias was limited because the villages were so small that the likelihood that all the children in the integrated method would have also been included by visiting the houses was very high, but for the other six villages, a selection bias was likely introduced because up to 62% of the sample was selected by convenience only and those persons would maybe not have been included if the sample was collected randomly.

This study shows a novel integrated mapping protocol to determine whether thresholds for public health interventions have been reached. The approach is logistically practical, cost-efficient and flexible. For schistosomiasis, our approach also results in more targeted MDAs compared with the district level implementation currently adapted by most integrated NTD program. The protocol uses mainly the age-specific disease indicators as recommended by WHO. Further, it sets the stage for all of the following integrated program activities essential for NTD elimination and control. Based on the lessons learned from the implementation in the first two countries and feedback we received form NTD colleagues, we do recognize that this novel mapping approach requires some modification to ensure that the most useful data are collected. For this reason, we are currently field testing an adapted protocol with all villages selected randomly among other minor changes.

## References

[1] Hotez PJ, Molyneux DH, Fenwick A, Kumaresan J, Sachs SE (2007). Control of neglected tropical diseases.. N Engl J Med.

[2] Musgrove P, Hotez PJ (2009). Turning neglected tropical diseases into forgotten maladies.. Health Aff (Millwood).

[3] Lammie PJ, Fenwick A, Utzinger J (2006). A blueprint for success: integration of neglected tropical disease control programmes.. Trends Parasitol.

[4] Grepin KA, Reich MR (2008). Conceptualizing integration: a framework for analysis applied to neglected tropical disease control partnerships.. PLoS Negl Trop Dis.

[5] Hotez PJ (2009). Mass drug administration and integrated control for the world's high-prevalence neglected tropical diseases.. Clin Pharmacol Ther.

[6] WHO (2006).

[7] Savioli L, Organization WH (2006). Neglected Tropical Diseases: Hidden successes, emerging opportunities..

[8] Baker MC, Mathieu E, Fleming FM, Deming M, King JD (2010). Mapping, monitoring, and surveillance of neglected tropical diseases: towards a policy framework.. Lancet.

[9] King JD, Eigege A, Richards F, Jip N, Umaru J (2009). Integrating NTD mapping protocols: Can surveys for trachoma and urinary schistosomiasis be done simultaneously?. Am J Trop Med Hyg.

[10] Ministere de la Sante M, Sante DNdl (2007). Plan Strategique de Lutte Contre Les Maladies Tropicales Negligees (MTNs)..

[11] Weil GJ, Lammie PJ, Weiss N (1997). The ICT Filariasis Test: A rapid-format antigen test for diagnosis of bancroftian filariasis.. Parasitol Today.

[12] WHO (2006). Trachoma control: a guide for programme managers..

[13] World Health organization (1991). The control of schistosomiasis..

[14] Katz N, Chaves A, Pellegrino J (1972). A simple device for quantitative stool thick-smear technique in Schistosomiasis mansoni.. Rev Inst Med Trop Sao Paulo.

[15] World Health Organization (2006). Preventive chemotherapy in human helminthiasis.

[16] Gyapong JO, Remme JH (2001). The use of grid sampling methodology for rapid assessment of the distribution of bancroftian filariasis.. Trans R Soc Trop Med Hyg.

[17] WHO (2005).

[18] Sturrock HJ, Picon D, Sabasio A, Oguttu D, Robinson E (2009). Integrated mapping of neglected tropical diseases: epidemiological findings and control implications for northern Bahr-el-Ghazal State, Southern Sudan.. PLoS Negl Trop Dis.

[19] Sturrock HJ, Gething PW, Clements AC, Brooker S (2010). Optimal survey designs for targeting chemotherapy against soil-transmitted helminths: effect of spatial heterogeneity and cost-efficiency of sampling.. Am J Trop Med Hyg.

[20] Ezz al Arab G, Tawfik N, El Gendy R, Anwar W, Courtright P (2001). The burden of trachoma in the rural Nile Delta of Egypt: a survey of Menofiya governorate.. Br J Ophthalmol.

[21] Faye M, Kuper H, Dineen B, Bailey R (2006). Rapid assessment for prioritisation of trachoma control at community level in one district of the Kaolack Region, Senegal..

[22] Paxton A (2001). Rapid assessment of trachoma prevalence–Singida, Tanzania. A study to compare assessment methods.. Ophthalmic Epidemiol.

[23] Fenwick A, Webster JP (2006). Schistosomiasis: challenges for control, treatment and drug resistance.. Curr Opin Infect Dis.

